# A prosocial fake news intervention with durable effects

**DOI:** 10.1038/s41598-023-30867-7

**Published:** 2023-03-09

**Authors:** Gábor Orosz, Benedek Paskuj, Laura Faragó, Péter Krekó

**Affiliations:** 1 ULR 7369-URePSSS-Unité de Recherche Pluridisciplinaire Sport Santé Société, Sherpas, Université de Lille, Université d’Artois, Université du Littoral Côte d’Opale, Liévin, France; 2 Department of Psychology, University College London, London, UK; 3 Institute of Psychology, ELTE Eötvös Loránd University, Budapest, Hungary; 4 Political Capital Institute, Budapest, Hungary

**Keywords:** Human behaviour, Statistics

## Abstract

The present online intervention promoted family-based prosocial values—in terms of helping family members—among young adults to build resistance against fake news. This preregistered randomized controlled trial study is among the first psychological fake news interventions in Eastern Europe, where the free press is weak and state-sponsored misinformation runs riot in mainstream media. In this intervention, participants were endowed with an expert role and requested to write a letter to their digitally less competent relatives explaining six strategies that help fake news recognition. Compared to the active control group there was an immediate effect (*d* = 0.32) that persisted until the follow-up four weeks later (*d* = 0.22) on fake news accuracy ratings of the young, advice-giving participants. The intervention also reduced the bullshit receptivity of participants both immediately after the intervention and in the long run. The present work demonstrates the power of using relevant social bonds for motivating behavior change among Eastern European participants. Our prosocial approach with its robust grounding in human psychology might complement prior interventions in the fight against misinformation.

## Introduction

To date, psychological interventions to arm people against misinformation have largely followed three strategies encouraged people to consume news with higher vigilance using nudges^1–6^, reduced susceptibility to misinformation by informing them about how they can be misinformed (inoculation^7–20^), or focused on developing digital media literacy^8,14,15,17,19,21–25^. The first strategy encourages more deliberative news consumption, which is very important in an age when the majority of people consume news through social media^26^ that deliberately serves self-affirming content to users to increase the time spent on the platform^27^. When the creation of echo chambers aligns with the financial-profit motive of all-powerful tech giants, trying to make people double-check sources and content is only laudable. However, fleeting nudges placed next to news content are hardly enough to create lasting change in people’s news consumption. Nudge interventions are not effective for all social groups^28^, and their long-term effect has rarely been investigated in prior studies^29^. The two latter strategies aim to develop digital skills and competencies, which is also a crucial endeavor in times when millions are coming online every day and consuming news without ever having experienced print journalism and/or content from outlets with robust editorial practices. In an environment where content from troll farms and that of leading news sites compete for readers’ attention, consumers need to be aware of typical manipulation strategies. Yet, the question remains: nudges alone can be enough to sustain behavior change in the long-term? There is very scant evidence for the long-term effects of digital literacy and inoculation interventions^12,13,20,22,25^ as the effects seem to quickly fade. Among these inoculation interventions, to our best knowledge, only Maertens et al.^13^ demonstrated a durable effect with a reminder that was demonstrated after three months. The present work aims to demonstrate long-term effects without a reminder and with a novel and different method that can potentially complement prior nudging and inoculation strategies.

Various motivational sources can be present to spot misinformation. We suggest that besides inoculation techniques, prosocial motivations might provide an additional layer of motivation to spot misinformation (e.g.^30,31^) compared to those, that predominantly build on individual intellect-related drivers (e.g.^16^). We root our psychological intervention in our post-socialist Hungarian sample's family-based prosocial motives.

## Cultural and historical context of family-based prosocial motivations

Hungary’s value structure is similar to its post-socialist neighbors since the first measurement in the 1960s^32–34^. Family, security, and home gained central importance in the lives of Hungarians during the transition into socialism after WW2 and into the 1950s—a period characterized by the socialist state party’s increasing control over public and private life when more than 90% of clubs, unions, and organizations not under direct political control were banned^35^. After the 1956 uprising the regime strived to provide higher living standards in exchange for societal depoliticization (see Gulyásszocializmus), and effectively a retreat into private family life. By 1982 more than four in five Hungarians said they “would not sacrifice themselves for anything besides their family”—the corresponding figures for Ireland, Denmark, and Spain were 55%, 49%, and 38%, respectively^35^.

During the economically turbulent times of the democratic transition in the 1990s, the importance of financial security and uncertainty avoidance was appreciated, but this did not diminish the significance of the family—from 1978 to 1998 the family’s safety was always the first or second most important value endorsed by Hungarians^36^. To this date, Hungarian society has not recovered from the disintegration of social ties precipitated by the socialist regime and further leveraged for myopic political motives by political forces in recent decades. The present research tried to harness these values to engender positive outcomes for the collective—using prosocial motivations based on familial ties to make youth more vigilant in the face of misinformation.

## Prosocial motives to identify misinformation

If political profiteering successfully capitalizes on these family-related values, why could not a psychological intervention encourage the recognition of misinformation does the same? Research shows that prosocial motivation -compared to egoistic- can drive people to work harder, smarter, safer, and more collaboratively (e.g.^30,37^). In hospitals, healthcare professionals were significantly more likely to appropriately follow hand hygiene practices when reminded of patients’ safety, compared to personal safety^38^. In a school setting, students performed better in boring and monotonous tasks, when it was for prosocial motives^31,39,40^. Prior misinformation intervention studies mainly promoted individual characteristics such as individual analytic thinking^4,5^ (e.g., being “smart” to spot fake news) and did not focus on motivations deriving social forces as a good reason to identify misinformation. Considering the historical and current political background promoting family values, in the Hungarian context we supposed that narrow prosocial motivations (e.g., helping a digitally incompetent family member) are the primary source of being motivated to spot fake news instead of broad prosocial motivations (e.g., helping a stranger). Similar to prior studies, in which prosocial motivations made students learn more persistently boring and tedious tasks (i.e. and leading to better grade point average months later), or prosocial reasons (i.e. protecting the health of their patients) made healthcare professionals clean their hands more often compared to the case when they had *only individual motivations without prosocial ones*, we expected that Hungarians equipped with narrowly family-based prosocial reasons will be persistently motivated to spot fake news.

These prosocial values went hand in hand with the digital competence-based position of the young adults in their families. We designed this intervention for young adults whose everyday practices involve a lot of online activities. Therefore, we supposed that they see themselves as more competent and feel more comfortable in the online world than their parents or grandparents. This social relation could put these young adults into an empowered position from which they can give authentic advice, especially after reading the six testimonials. These testimonials were not supposed to trigger reactance as they were labeled as mere opinions about the presented strategies. The six quotes also served as core content and a channeling towards the indirect self-persuasive, saying-is-believing exercise in which the participants could write a letter about the misinformation identification strategies.

## Psychological mechanisms besides prosocial values

In contrast to other psychological interventions that implicitly place participants in a role of relative incompetence or ignorance, our approach consciously tried to capitalize on participants’ self-enhancement motives^41^ by affording them the role of a digital expert in their families—largely due to their young age that can help more elderly family members ^42^. Vigilance for online misinformation was presented as a behavior they could both role model and explicitly pass on to the generations of parents and grandparents. On top of their intergenerational digital advantage, we also wanted to tap into a potentially more relevant source of status concerns, the peer group, hence also described how digital responsibility can garner respect and status in their generation too^43^.

Both the treatment and the control materials were framed in a learning mindset (see ^44^), meaning that digital behaviors were presented as competencies that can be developed through (1) effort, (2) elaborate learning strategies, and (3) advice from those with more competence. The backbone of the intervention material was a selection of strategies^22^ that help people navigate online news and spot misinformation. Participants learned about these strategies through testimonials from peer group members.

Some testimonials used *self-distanced self-talk* to open up space between experiential and analytical facets of the self^45^. For instance, the ‘Questioning information that is outrageous’ strategy’s presentation included the following excerpt: “*I used to be pretty scared about all sorts of things I read online. For this reason, I have figured out that if a news story comes across with some threatening message, I will stop for a moment and ask myself: John, can this hurt you now, or is it unfounded nonsense that was written just to make you be scared?*”*.* With this strategy, we did not only wish to encourage participants’ analytical thinking to flag suspect information, but also to mitigate the potentially fear-inducing features of misinformation by enabling its perusal from a safe mental distance.

The testimonials were followed by a *self-persuasive exercise* similar to the prosocial purpose intervention of Yeager et al.^31^. Composing a letter to their older family members, participants were expected to indirectly persuade themselves—an approach that can yield more lasting effects than direct persuasion^46^. Writing the letter and advocating for strategies the author does not follow can highlight the distance between their advice and their behavior and therefore induce hypocrisy concerns, which can be another motivator for aligning their behavior with their advice^47,48^.

## Fake-news interventions, scalability, and long-term effects

While various intervention approaches tackle misinformation, only a few of them find long-term effects^12,13,20,22,25^. Social media platforms like to experiment with accuracy nudges, due to their brevity, low cost, and scalability on social media^1–6,49^. Yet, such nudges are deeply tied to situational cues, hence the present, and do not tap into deeper motivations or a wider narrative about news consumption^50^. So even if accuracy reminders preceding headlines effectively lead to increased truth discernment as evidenced by sharing intentions^4,5^; however, it is not necessarily the case when these cues are absent. We argue that nudges remain a double-edged sword as they are effective immediately but most of the time, they fail to produce long-term, recursive effects^51,52^.

Inoculation^7–20^ and other forms of digital media literacy interventions^8,14,15,17,20–25^ rarely produce reliable long-term effects. Their gamified and explicitly educational formats require cooperation and in-depth engagement^50,53^, which can limit their scalability^49^ and sometimes they take several hours, which makes them difficult to implement among the general public—through more recent attempts translating these approaches to video formats may address these issues^16^. Unfortunately, their immunizing effects can become significantly smaller over time (e.g., effect sizes dropped from *d* = 0.13 to *d* = 0.05 in India^22^), or they can decrease considerably over a short period (e.g., effect sizes dropped from *d* = 0.95 to *d* = 0.28 within one week and from *d* = 0.20 to *d* = 0.08 in the US^12,13,22^.) In sum, the efficacy of scalable misinformation interventions on accuracy or discernment rating fades quickly. By using the persuasive power of prosocial motivations, we aimed to mobilize deep-seated social motivations that facilitate sustained behavior change over time and that can complement prior strategies to cope with systemic disinformation campaigns in an informational autocracy.

## Methods

### Participants

#### Preregistered plans

Based on the preregistration, we aimed to gather data among high school students, university students, and in a comprehensive sample that is close to representative of Hungarian adults. Data collection among high school students was challenging as political fake news content was impossible to use (for legal reasons deriving from the present, illiberal political context), the COVID-19 pandemic made it difficult to gather data and reduce attrition, and teachers and students were not sufficiently incentivized to provide reliable data (we could not pay them for legal reasons). For the comprehensive sample aiming to draw 800–1200 respondents from the general population, we asked five polling companies and none of them could guarantee even a 40% attrition rate regardless of the incentive structure. We, therefore, opted out of data collection with these companies. In the university sample, our goal was to recruit at least 787 participants (with *d* = 0.2, alpha error prob = 0.05, power = 0.8, and a number of predictors = 1, based on Guess et al.^22^) to be able to test our main hypothesis about fake news accuracy.

### Participants of the study

**Figure 1:**
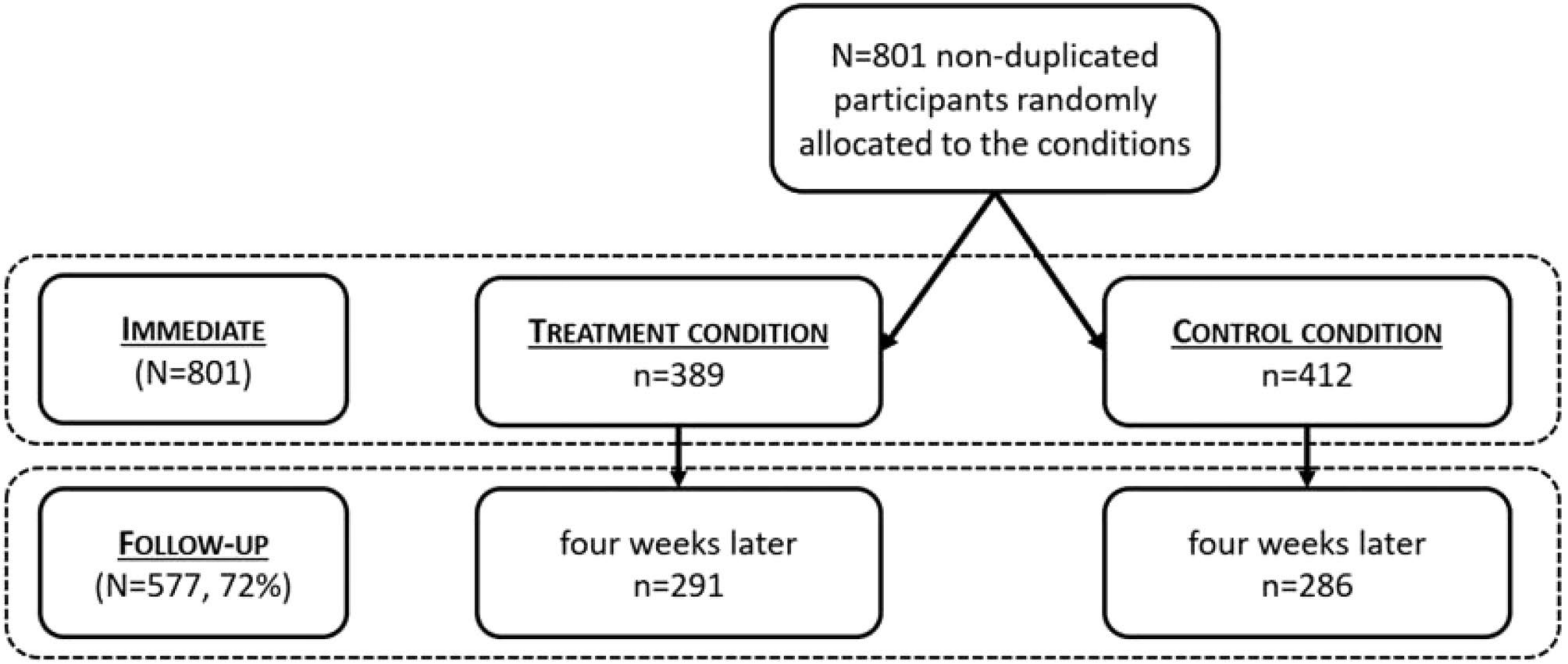
Sample characteristics and attrition.

Young adults participated from a Hungarian public university with various majors took a credit course in which participation was voluntary. Among them, 801 reached the randomized intervention or control materials (*M*_age_ = 22.02; *SD*_age_ = 4.11; 73.46% female; 95.33% Caucasian, 34.40% first-generation). We found this form of data gathering and sampling the most suitable as polling companies could provide similar samples with much higher expected attrition. There was some attrition in the follow-up, as students did not provide an appropriate Student ID which prevented us from matching their follow-up responses to their intervention data. This led to an attrition of 27.72% of students with intent-to-treat follow-up data from 72.28% of the allocated students (*N* = 577, *M*_age_ = 21.98; *SD*_age_ = 3.85; 76.08% female; 96.55% Caucasian, 33.94% first-generation, for a summary see Fig. 1). In sum, though the number of participants in the follow-up was somewhat smaller than expected, the overall attrition was not high.

### Procedure

This study was conducted with the ethical approval of Eötvös Loránd University’s institutional review board, in accordance with the Declaration of Helsinki, and with the informed consent of the participants. After consenting, participants first filled out the “bullshit” receptivity scale ^54^, demographics, and then proceeded to their randomly assigned condition.

**Figure 2:**
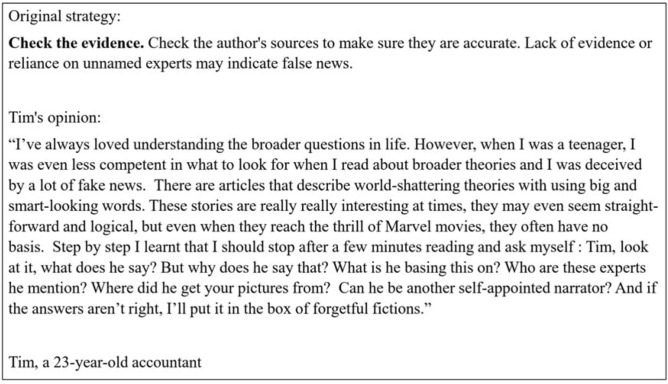
A testimonial explaining one of the six strategies. First, participants could read six testimonials explaining six strategies.

In the *intervention material*, the survey was framed as a contribution to an online media education program targeting parents’ and grandparents’ generation. First, participants reviewed six scientifically supported strategies (all adapted from Guess et al.^22^) explained through peer testimonials to spot online misinformation (skepticism for headlines; looking beyond fear-mongering; inspecting the source of news; checking the evidence; triangulation; considering if the story is a joke; see an example in Fig. 2). Based on prior research ^31,55,56^, these testimonials were supposed to provide normative information about other students’ negative experiences of not spotting misinformation and positive experiences of identifying misinformation. In contrast to prior studies, we used quotes as narratives, but we did not use the descriptive norms in a numeric (e.g., XY% of the students) format such as in the intervention of Yeager et al.^31^.

**Figure 3:**
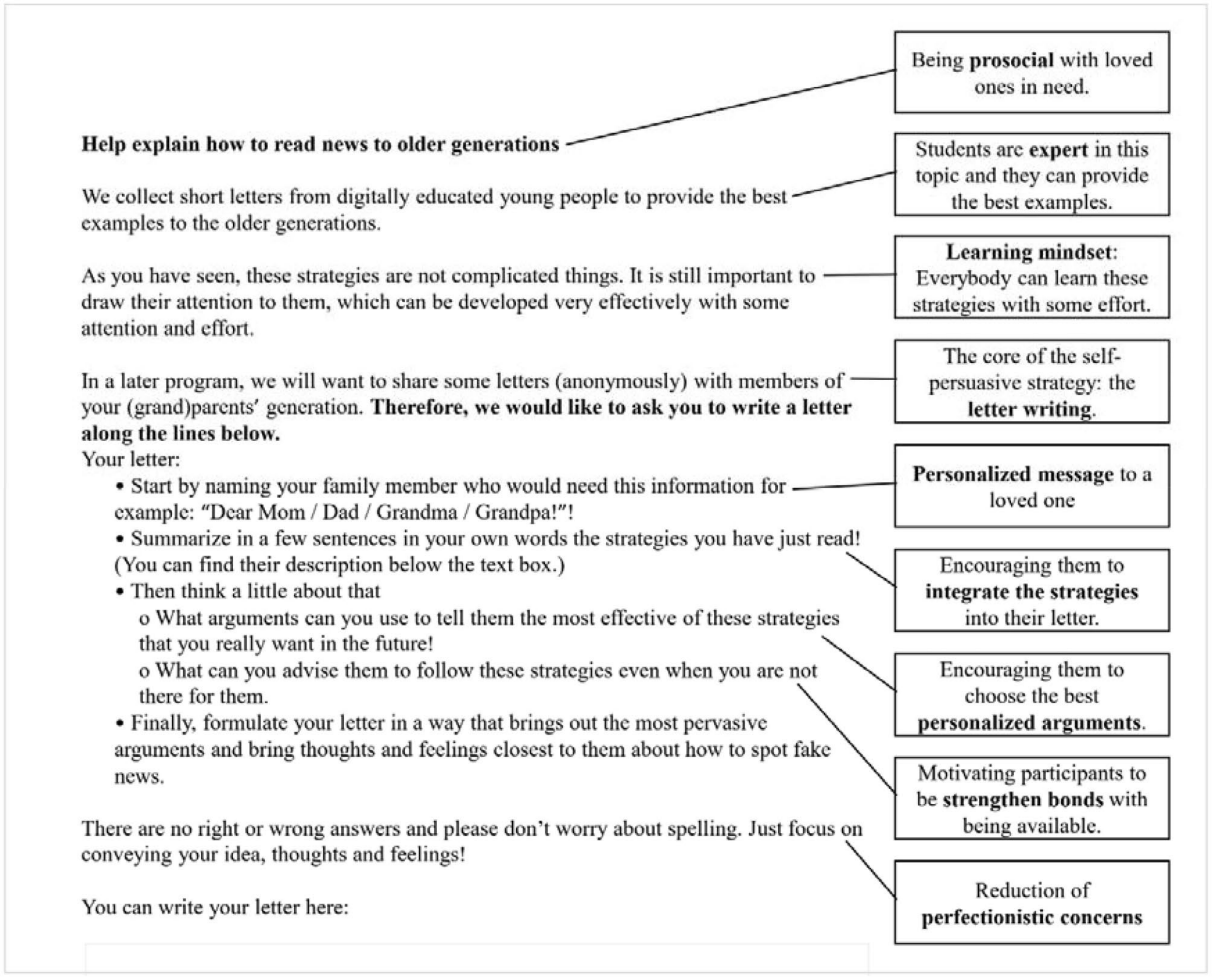
Psychological mechanisms of the self-persuasive message. After reading the six testimonials, participants wrote a letter to a loved one in which they explained the strategies.

**Figure 4:**
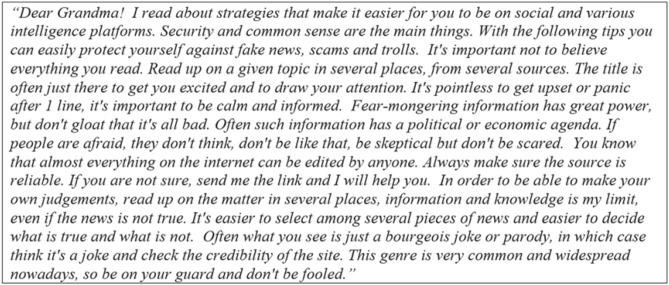
A randomly chosen letter following the above-described instructions (translated by DeepL).

Second, they were requested to write a brief letter to a close family member in which they summarize the six strategies and were asked to reflect on the best arguments and advice that would convince their relatives to implement these strategies in their everyday life (for the psychological mechanisms and the instruction see Fig. 3, for a randomly chosen letter see Fig. 4.).

The structure of the *control condition* was very similar to the intervention; however, the topic of fake news did not appear in the control materials as it was framed as an advice-giving task about how to use social media sites like Facebook appropriately. It described practices of older generations that young adults find awkward and provided advice for avoiding these embarrassing behaviors. In the materials, participants found six examples of implicit norm violations of social media use (mixing up private messaging with Facebook’s feed; sending virtual flowers on the wall for birthdays; incorrect use of emojis; uploading inappropriate profile pictures; anomalies during video chat; sending inappropriate invites to online games). Subsequently, similar to the treatment, they were asked to compose a letter to an elderly relative summarizing these practices and to detail the best advice for avoiding these social media behaviors. In sum, the content of the control materials was related to appropriate behaviors in social media sites without referring to news content, fake news, and misinformation.

**Figure 5:**
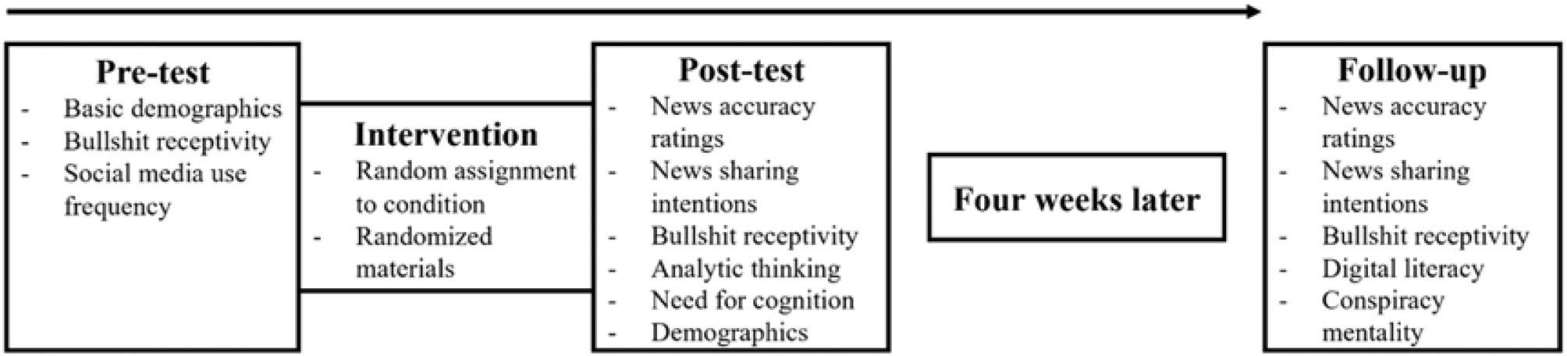
Timeline.

The timeline of our study can be seen in Fig. 5.

### Measures

*Fake news accuracy* To assess fake news accuracy ratings, we used the protocol of Pennycook and Rand^57^ by asking participants to evaluate the accuracy of eight carefully pretested and culturally adjusted real and eight fake news items (half of them with political content and half of them with apolitical content) on four-point scales (not at all accurate/not very accurate/somewhat accurate/very accurate). Based on the preregistration (https://osf.io/8tgk6), in the present analysis, we focused on fake news accuracy scores as outcome variables; however, we used real news accuracy ratings as covariates in the robustness analyses. The fake and real news headlines were different in the post-intervention material and the follow-up and they can be found online in the Supplementary Materials.

*Fake and real news sharing intentions* were measured post-intervention using the scale of Pennycook and Rand^57^ separately for each news item: “*Would you consider sharing this story online (for example, through Facebook)*?” with four response options: *I never share any content online, no, maybe, yes.*

*Bullshit receptivity* was assessed with ten items^54^ such as “*Interdependence is rooted in ephemeral actions*”*.* Respondents filled out half of the scale pre-intervention, the other half post-intervention, and the full scale again in the follow-up. The response scale ranged from 1 (not at all profound) to 5 (very profound). All the pre- and post-intervention and follow-up measures’ internal consistency was excellent (⍶_pre_ = 0.84; ⍶_post_ = 0.80; ⍶_follow-up_ = 0.90).

*Conspiracy mentality* was measured in the follow-up with the CMQ^58^ with five items such as “*I think that many very important things happen in the world, which the public is never informed about*”. Respondents rated their agreement with the statements using percentages ranging from 0% (*coded as 1*) to 100% (*coded as 11*) with steps of 10%. The internal consistency of the measure was borderline (⍶_follow-up_ = 0.69).

*Digital literacy* was measured in the follow-up with five items^59^ such as “*I rely on family members to introduce me to new technology*”. Respondents indicated how often these statements applied to them using a scale from 1 (*never*) to 5 (*very often*). The reliability of the scale was acceptable (⍶_follow-up_ = 0.70).

*Need for cognition* was assessed post-intervention using five items from the NFC scale^60^ such as *“It's enough for me that something gets the job done, I don't care how or why it works”* (reversed item). Respondents rated their agreement with the statements with a scale ranging from 1 (*I do not agree at all*) to 7 (*I totally agree*). The reliability of the scale was acceptable (⍶_pre_ = 0.75).

*Cognitive reflection* was measured post-intervention using the CRT^61,62^ with five items such as: *“A bat and a ball cost $1.10. The bat costs $1.00 more than the ball. How much does the ball cost?”* Cognitive reflection items were coded as correct (1) and wrong (0).

*Socioeconomic status* was measured by ethnic minority status and parental level of education in terms of first-generation (none of the parents had tertiary education) vs. continuing-generation (at least one of the parents had tertiary education) status.

### Analytic strategy

Participants of the intervention condition (contrasting to the control) were expected to provide less correct ratings on fake news (H1), and they would intend to share fake news to a smaller extent (H2), even after controlling for relevant individual differences (H1a, H2a). We also expected that fake news accuracy ratings would be negatively related to analytic thinking (H3), and need for cognition (H6); but positively to bullshit receptivity (H4), digital literacy (H5), socioeconomic status (parental education and minority status, H7), conspiracy mentality (H8), and conservative party voters (H9). Finally, we expected that treatment (contrasting to control) would lead to lower levels of bullshit receptivity for the immediate and the longer-run results (one-month follow-up, H10).

Based on the preregistration, for the two main hypotheses, we use OLS regression analyses to measure the effect of the condition on fake news accuracy (H1) and sharing intention (H2). Subsequently, we control for relevant (correlated) individual differences and assess the effect of the treatment above and beyond these individual differences. We run these analyses for the immediate and the longer-run results (one-month follow-up). OLS regression analyses and correlations are used to assess the relationship between the above-mentioned individual differences (H3-H8) and fake news accuracy ratings as well as sharing intentions. With an OLS regression analysis, we assess the effect of the treatment on bullshit receptivity scores after controlling for pre-intervention bullshit scores (H10). We run these analyses for the immediate and the longer-run results (one-month follow-up).

## Results

### Preliminary analyses and attrition

We examined overall attrition (independent of condition) and differential attrition (condition-dependent) in both samples along sociodemographic (age, gender, residence, parental level of education, first-generation status), and relevant psychological characteristics (“bullshit receptivity”, need for cognition, and analytic thinking).

Considering the *overall* attrition, we found that the proportion of male (*p* = 0.004) and minority students (*p* = 0.003) was significantly reduced compared to the baseline. We did not find differences in any other sociodemographic, social media use, or psychological variables between those who participated in the follow-up and those who dropped out. Finally, in terms of *differential attrition*, students retained, as compared to those not retained, did not differ significantly except for gender. We found that the proportion of female participants was higher in the control condition than in the treatment (*z* = −2.10; *p* = 0.036).

Considering the above-mentioned individual differences, there were no baseline differences between the treatment and the control groups (*p* > 0.13). The proportion of those people who support the government was marginally higher in the control condition (*p* = 0.073). Descriptive statistics of the main variables can be found online in the Supplementary Material (Table S1 and Table S2). In addition, 78.6% of the participants followed the instructions appropriately in terms of writing about the strategies they read about previously and giving advice to their relatives and participants spent 10.18 min on average writing the letter. The proportion of participants who did not follow the instructions were very similar in the treatment (21.56%) and the control (21.21%) conditions, and they did not differ significantly (*p* = 0.907).

### Primary analyses

#### Short- and long-term fake news accuracy ratings (H1)

**Figure 6:**
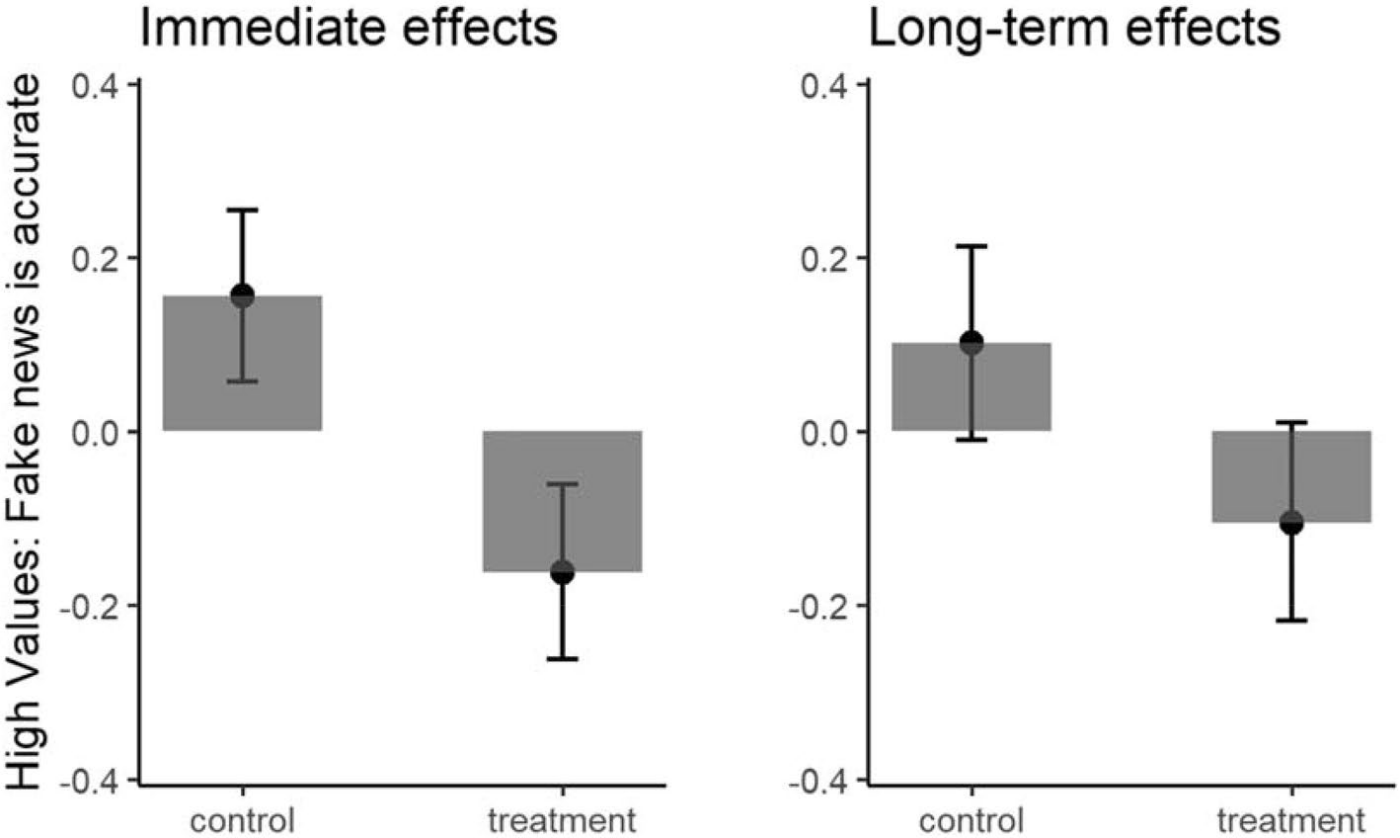
Accuracy evaluation of fake news immediately after the intervention (left panel) and 4 weeks later (right panel). Error bars represent standard errors. The y-axis represents standardized scores in terms of fake news accuracy ratings controlled for real news accuracy ratings. Higher scores indicate that fake news is accurate.

Overall, the results showed that the intervention produced significant immediate, *b* = 0.16, *t*(689) = 4.30, *p* = 0.001, *d* = 0.32, and long-term (one-month follow-up) accuracy rating improvements, *b* = 0.09, *t*(576) = 2.62, *p* = 0.009, *d* = 0.22, relative to the control condition (see Fig. 6). The immediate effects held above and beyond relevant individual differences in real news ratings, demographic (age, gender, parental education, and minority status), and relevant analytical thinking (cognitive reflection and need for cognition) variables *b* = 0.15, *t*(677) = 4.28, *p* < 0.001, *d* = 0.30. The long-term effects also held in models after controlling for all the above-mentioned variables plus digital literacy and conspiracy mentality *b* = 0.07, *t*(538) = 2.11, *p* = 0.036, *d* = 0.17. (We excluded these variables from the model reported for immediate results to mitigate the attrition associated with them—as the last measures captured in the survey missing data was highest for these items). Including these two control variables, although decreased the power of the models for immediate effects did not change the significant effect of the treatment on fake news accuracy ratings, *b* = 0.12, *t*(538) = 3.17, *p* = 0.002, *d* = 0.24.) Considering the fake news accuracy scores as outcomes, the time × condition interaction was not significant with (*b* = 0.14,* t* = 1.58, df = 548, *p* = 0.116) or without control variables (*b* = 0.13, *t* = 1.54, df = 548, *p* = 0.124). It means that over time the effect of the intervention on fake news accuracy did not decrease significantly with or without considering other relevant control variables and also if we considered only those participants who followed the instructions.

#### Short- and long-term fake news sharing intentions (H2)

The treatment produced neither immediate nor long-term changes in sharing intentions relative to the control condition (all *p*s > 0.367).

#### Bullshit receptivity (H10)

**Figure 7:**
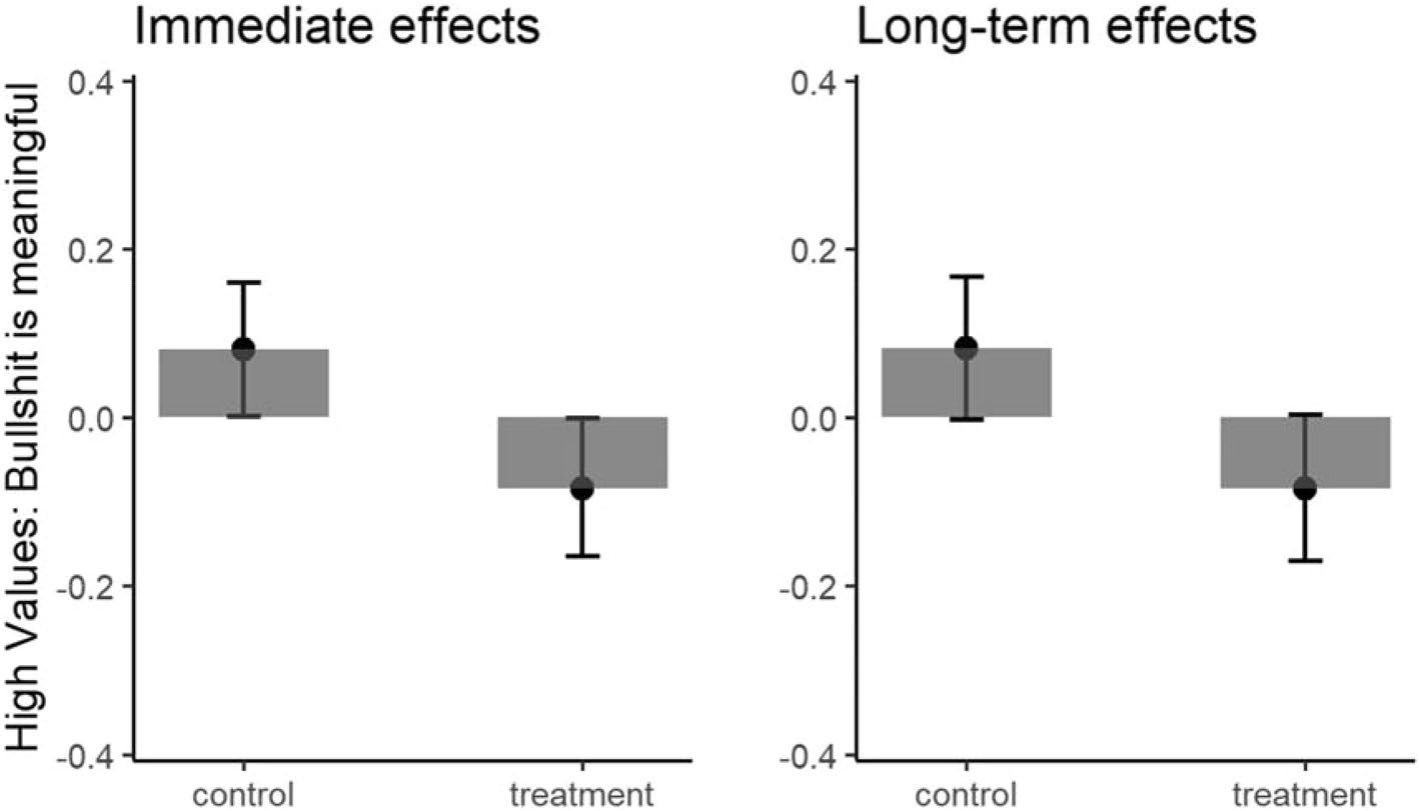
Bullshit receptivity immediately after the intervention (left panel) and 4 weeks later (right panel). Error bars represent standard errors. The y-axis represents standardized scores in terms of bullshit receptivity ratings controlled for pre-intervention bullshit receptivity ratings. Higher scores indicate beliefs in bullshit are meaningful such as “interdependence is rooted in ephemeral actions”.

It showed that the intervention produced significant immediate, *b* = 0.13, *t*(688) = 2.82, *p* = 0.004, *d* = 0.16, and long-term (1-month follow-up) accuracy rating improvements, *b* = 0.14, *t*(575) = 2.67, *p* = 0.008, *d* = 0.17, relative to the control condition (see Fig. 7).

### Secondary analyses

Participants with strong analytical skills (high scores on cognitive reflection task, H3; *β* = −0.15, *t*(687) =  −4.21, *p* < 0.001), lower levels of receptivity to bullshit information (H4;* β* = 0.23, *t*(688) = 6.42, *p* < 0.001), high levels of digital literacy (H5;* β* = 0.21, *t*(548) = 5.26, *p* < 0.001), a strong need for cognition (H6; *β* = −0.17, *t*(687) =  −4.63, *p* < 0.001), a high socioeconomic position defined by majority status (H7;* β* = −0.42, *t*(687) =  −2.45, *p* < 0.001), but not by the parental level of education (p = 0.957), low levels of conspiracy mentality (H8; *β* = 0.24, *t*(548) = 6.13, *p* < 0.001) evaluated fake news less accurately when real news evaluation was controlled for.

Finally, we explored the moderating effect of the relevant individual differences and found neither short-term, nor long-term moderation for analytic thinking, bullshit receptivity, digital literacy, need for cognition, socioeconomic status, gender, or conspiracy mentality (all *p*s > 0.064). Conspiracy mentality marginally moderated the treatment’s effect on fake news accuracy ratings immediately after the intervention, *β* = −0.14, *t*(546) =  −1.86, *p* < 0.064.

## Discussion

This research was conducted in Hungary, an Eastern European country that has been experiencing “democratic backsliding” in the past decades^63,64^. The systemic disinformation campaigns orchestrated by the illiberal Hungarian government^65,66^, and Russia’s soft influence in social media^67^ using online astroturfing techniques^20^ pose danger to democratic institutions. The context, therefore, has relevance, as governmental propaganda is neither an eminent promoter of analytical thinking in general nor of critical news consumption in particular. Yet, despite its enormous pertinence, no psychological fake news intervention has been conducted in this country before. As described above, there is also a handful of scientific studies^12,13,20,22,25^ that have assessed and demonstrated the long-term effects of misinformation interventions.

Our family-oriented prosocial wise intervention was tested among Hungarian young adults with a one-month follow-up via a behavioral fake news recognition task. We observed, as hypothesized in the preregistration, short-term effects for recognizing (but not sharing) fake news (*d* = 0.32) and also a long-term effect (*d* = 0.22) compared to the control group. Although our sample was not representative, the present results confirm that using Guess et al.’s digital literacy^22^ tips in combination with the principles of wise social psychological interventions^51^ can produce long-term changes in misinformation recognition in a cultural context that is dissimilar to the US or India. These short- and long-term effects were present after taking into account real news accuracy ratings, and relevant sociodemographic, digital, and psychological individual differences, and the decrease in the effect was not significant over a month. Another preregistered collateral benefit, the reduction in bullshit receptivity, was also observed both in the short- and the long run. All in all, the intervention had a durable impact on the recognition of fake news and receptivity to “bullshit”; but it did not change fake news sharing intentions in the short- or the long-term. One of the reasons for this unexpected result might be that sharing judgments do not always follow accuracy ratings: users often share news independently from their perceived truthfulness^4,5,49^. Furthermore, the sharing intention of any sort of news was very low in the present sample, which can also explain why our intervention did not change sharing intentions.

### Theoretical and practical implications

The novelty of the present intervention is that it neither nudged people to spot fake news^1–6^ nor did it explicitly motivate them to build digital competences^8,14,15,17,19,21–25^ or inoculate people with certain skills to approach the news with more suspicion^7–20^. While these studies mainly focused on the individual cognitive capacities of news consumers, we turned toward social motivations. Capitalizing on prior studies^31,68^ to promote *prosocial motivations* among young adults, and aiming to change the *reasons and the meaning of why fake news detection can be (pro)socially important* to them*.* Instead of putting participants in the inferior position of someone in need of learning to avoid incompetence, we addressed them as digital experts who can contribute to the digital competency of their loved ones. This might not only increase the competency of young adults through the ‘saying-is-believing’ exercise^46^, but it also musters two distinct persons’ cognitive capacities to interpret dubious news content. Such interventions can open the door to intergenerational discussions through which younger and older people can become better armed against misinformation.

Another important note relates to the *cultural context*, in which we did not arbitrarily pick family-related prosocial values. Studies by Aronson (see^46^) integrated the ‘saying-is-believing’ technique into a family context where an older sibling advised a younger one and not into a general social context where, for example, a student advises a schoolmate^44^. We supposed that the proximity between the advisor and advisee can enhance the persuasiveness of the message. The other reason was that in Hungary, family- and security-related values have been reported as the most important to respondents since their first measurement. The focus on one’s narrow communities in Hungarian society has deep historical roots and the sociological literature has developed a strong consensus on the dominance of communal prosocial goals involving family and close friends over distal and broader societal goals^32,34^. The present work demonstrates how it is possible to use these psychological forces to make people more vigilant in the face of misinformation and we expect that this family orientation might be a potentially useful persuasion technique in other Eastern European, semi-traditional, or ingroup-collectivist countries.

### Limitations and future directions

Despite being a preregistered, randomized controlled trial, our study has some limitations that need acknowledgment. First, we recruited participants from educational institutions so our sample is not representative of the Internet-using population: future studies should strive to recruit people outside tertiary education—a group that is more prone to fall for misinformation^69^. Second, we used Pennycook and Rand’s measure^57^ of the dependent variable instead of capturing actual social media behaviors over time. Third, even if the present study has a great potential for scalability, long-term effects can be hardly expected if participants do not take approximately ten minutes and invest the energy to write such letters as can be seen in Fig. 4. In addition, very probably, not only the length but the quality of the letters might also matter. Future studies may want to analyze deeper qualitative data from the student letters. It is possible that students who provided more elaborate responses benefitted more in terms of fake news recognition. Finally, the main finding of the present intervention in terms of prosocial and social motivations can be beneficial to spot fake news and can be integrated into nudge- and inoculation-based studies. For example, similar to Maertens et al.^13^, brief prosocial reminders could also contribute to long-term effects. Promoting analytic thinking as a form of prosocial care for a family member is compatible with the inoculation intervention techniques and also with nudges. Therefore, nudges and inoculation interventions highlighting prosocial family values can be also beneficial in interdependent cultural contexts similar to the Hungarian one. These integrative studies might be the topic of further collaborations that use a broad variety of theoretical principles and applied techniques.

## Conclusion

Family-related prosocial values can open new horizons in the fight against misinformation in Eastern Europe. The present intervention is an example of leveraging family values to motivate people to use their cognitive capacities while consuming news in a context where mainstream media is a vector of seriously problematic news content. In the region, the Hungarian government is not the only one using public televisions, radios, and local news to regularly disseminate news with ambiguous content—more vigilance about news veracity could prove a brake in the slide back from democracy.

## Supplementary Information

Supplementary Tables.

Dataset S1.

## Data Availability

The dataset that supports the findings will be openly available on Open Science Framework (https://doi.org/10.17605/OSF.IO/VCF36) and it is submitted with the manuscript. The link to the preregistration can be found here: https://osf.io/8tgk6.
