# Supplementary material for: A prosocial fake news intervention with durable effects
Source: Sci Rep. 2023 Mar 9;13:3958. doi: 10.1038/s41598-023-30867-7 (PMC9996558; doi:10.1038/s41598-023-30867-7)
Supplement: Supplementary file 1 — Supplementary Tables. [file 41598_2023_30867_MOESM1_ESM.docx]

**Supplementary Materials**

**for**

**A Prosocial Fake News Intervention with Durable Effects**

Contents

[Table S1: Descriptive statistics of the main variables 2](#_Toc127645505)

[Table S2: Sharing frequencies of fake and real news 3](#_Toc127645506)

[Media truth discernment results 4](#_Toc127645507)

[Regression Tables for Hypotheses 1 and 2 5](#_Toc127645508)

# Table S1: Descriptive statistics of the main variables

|  | Intervention | | Control | |
| --- | --- | --- | --- | --- |
|  | *Raw M* | *SD* | *Raw M* | *SD* |
| Accuracy of fake news – immediately post-intervention | 1.81 | 0.49 | 1.97 | 0.48 |
| Accuracy of real news – immediately post-intervention | 2.49 | 0.43 | 2.50 | 0.41 |
| Accuracy of fake news – follow-up | 1.86 | 0.44 | 1.96 | 0.41 |
| Accuracy of real news – follow-up | 2.49 | 0.47 | 2.52 | 0.48 |
| Bullshit receptivity – pre-intervention | 2.42 | 0.91 | 2.37 | 0.89 |
| Bullshit receptivity – post-intervention | 1.98 | 0.81 | 2.08 | 0.82 |
| Bullshit receptivity – follow-up | 2.19 | 0.87 | 2.28 | 0.83 |
| Cognitive reflection – immediately post-intervention | 3.06 | 1.44 | 3.12 | 1.33 |
| Need for cognition – immediately post-intervention | 4.93 | 1.11 | 4.86 | 0.99 |
| Digital literacy – follow-up | 2.28 | 0.66 | 2.29 | 0.62 |
| Conspiracy mentality – follow-up | 66.10 | 14.70 | 68.10 | 14.84 |

*Note:* The acceptance of fake and real news was measured with a scale from 1 (not at all accurate)
to 4 (very accurate), higher means indicate higher perceived accuracy. Bullshit receptivity scales were measured using 5-point Likert scales (1 – not at all profound; 5 – very profound): the higher the mean, the greater bullshit receptivity is. Cognitive reflection items were coded as correct (1) and wrong (0), and a higher mean represents higher analytical thinking. We used 7-point Likert scales to measure the Need for cognition (1 – I don’t agree at all; 7 – I completely agree): the higher the mean, the greater the need for cognition. Answers to the Digital literacy items ranged between 1 (never) and 5 (very often), lower means indicate higher digital literacy. Conspiracy mentality was measured with a scale from 1 (low conspiracy mentality) to 11 (high conspiracy mentality): the higher the mean, the higher the conspiracy mentality is.

# Table S2: Sharing frequencies of fake and real news

|  | Intervention | | Control | |
| --- | --- | --- | --- | --- |
|  | *Raw M* | *SD* | *Raw M* | *SD* |
| Fake news sharing – immediately post-intervention | 0.20 | 0.77 | 0.20 | 0.75 |
| Real news sharing – immediately post-intervention | 0.37 | 1.01 | 0.29 | 0.86 |
| Fake news sharing – follow-up | 0.09 | 0.47 | 0.07 | 0.42 |
| Real news sharing – follow-up | 0.27 | 0.96 | 0.21 | 0.67 |

*Note:* Based on Pennycook and Rand (2019) the response options were: *I never share any content online (0), no (0), maybe (1), yes (2)*

# Media truth discernment results

For the sake of clarity, in line with prior studies, we ran analyses to identify the effect of the intervention on a common composite score called media truth discernment. This score can be calculated by subtracting fake news accuracy ratings from real news accuracy scores. According to the results, there was a significant treatment effect immediately after the intervention (*b*=0.15, *t*(685)=3.62, *p*<0.001, *d*=0.27), but a somewhat weaker effect was found in the long run (*b*=0.07, *t*(574)=1.39, *p*=0.165, *d*=0.12) although these effects are consistent with prior studies (see for example, Guess et al., 2020).

# Regression Tables for Hypotheses 1 and 2

H1: Participants of the intervention condition (contrasting to the control) were expected to provide less correct ratings on fake news.

|  | Fake news accuracy score  Immediate without control variables | | |
| --- | --- | --- | --- |
| *Predictors* | *Estimates* | *CI* | *p* |
| (Intercept) | -0.17 | -0.27 – -0.06 | 0.002 |
| Condition | 0.33 | 0.18 – 0.47 | <0.001 |
| Observations | 687 | | |
| R^2^ / R^2^ adjusted | 0.027 / 0.025 | | |

H1a: The treatment (contrasting to control) will lead to lower accuracy ratings of fake news immediately after the intervention even after controlling for those individual differences that are related to fake news accuracy ratings.

|  | Fake news accuracy rating  Immediate with control variables | | |
| --- | --- | --- | --- |
| *Predictors* | *Estimates* | *CI* | *p* |
| (Intercept) | 0.05 | -0.31 – 0.41 | 0.786 |
| Real news accuracy rating | 0.31 | 0.24 – 0.38 | <0.001 |
| Condition | 0.30 | 0.16 – 0.44 | <0.001 |
| Age | -0.01 | -0.09 – 0.06 | 0.706 |
| Gender | 0.25 | 0.08 – 0.41 | 0.003 |
| First-generation status | -0.03 | -0.17 – 0.12 | 0.706 |
| Minority status (ethnic) | -0.40 | -0.73 – -0.07 | 0.017 |
| Cognitive Reflection Task | -0.10 | -0.17 – -0.03 | 0.008 |
| Need for Cognition | -0.12 | -0.19 – -0.05 | 0.001 |
| Observations | 682 | | |
| R^2^ / R^2^ adjusted | 0.158 / 0.148 | | |

|  | Fake news accuracy rating  Immediate with control variables | | |
| --- | --- | --- | --- |
| *Predictors* | *Estimates* | *CI* | *p* |
| (Intercept) | 0.45 | -0.00 – 0.91 | 0.051 |
| Real news accuracy rating | 0.29 | 0.22 – 0.37 | <0.001 |
| Condition | 0.24 | 0.09 – 0.39 | 0.002 |
| Age | -0.01 | -0.09 – 0.08 | 0.832 |
| Gender | 0.08 | -0.12 – 0.27 | 0.439 |
| First-generation status | -0.05 | -0.21 – 0.11 | 0.563 |
| Minority status (ethnic) | -0.63 | -1.04 – -0.21 | 0.003 |
| Cognitive Reflection Task | -0.07 | -0.15 – 0.02 | 0.111 |
| Need for Cognition | -0.09 | -0.17 – -0.00 | 0.039 |
| Digital literacy | 0.14 | 0.05 – 0.22 | 0.002 |
| Conspiracy mentality | 0.19 | 0.11 – 0.27 | <0.001 |
| Observations | 547 | | |
| R^2^ / R^2^ adjusted | 0.203 / 0.188 | | |

H1: The treatment (contrasting to control) will lead to lower accuracy ratings of fake news in the long run (one-month follow-up).

|  | Fake news accuracy rating: long-term | | |
| --- | --- | --- | --- |
| *Predictors* | *Estimates* | *CI* | *p* |
| (Intercept) | -0.11 | -0.23 – 0.01 | 0.064 |
| Condition | 0.22 | 0.05 – 0.38 | 0.009 |
| Observations | 576 | | |
| R^2^ / R^2^ adjusted | 0.012 / 0.010 | | |

H1a: The treatment (contrasting to control) will lead to lower accuracy ratings of fake news for longer-run (one-month follow-up) even after controlling for those individual differences that are related to fake news accuracy ratings.

|  | Fake news accuracy score  Long-term with control variables | | |
| --- | --- | --- | --- |
| *Predictors* | *Estimates* | *CI* | *p* |
| (Intercept) | 0.26 | -0.22 – 0.75 | 0.284 |
| Real news accuracy rating | 0.23 | 0.15 – 0.32 | <0.001 |
| Condition | 0.17 | 0.01 – 0.33 | 0.036 |
| Age | -0.01 | -0.10 – 0.08 | 0.849 |
| Gender | 0.02 | -0.19 – 0.23 | 0.833 |
| First generation status | -0.13 | -0.31 – 0.04 | 0.129 |
| Minority status (ethnic) | -0.32 | -0.76 – 0.12 | 0.154 |
| Cognitive Reflection Task | -0.00 | -0.09 – 0.09 | 0.977 |
| Conspiracy mentality | 0.07 | -0.01 – 0.16 | 0.085 |
| Need for Cognition | -0.04 | -0.13 – 0.05 | 0.352 |
| Digital literacy | 0.20 | 0.11 – 0.29 | <0.001 |
| Observations | 547 | | |
| R^2^ / R^2^ adjusted | 0.117 / 0.101 | | |

H2: The treatment (contrasting to control) will lead to reduced sharing of fake news for the immediate and longer-run results (one-month follow-up).

|  | Fake news sharing intention – Immediate | | |
| --- | --- | --- | --- |
| *Predictors* | *Estimates* | *CI* | *p* |
| (Intercept) | -0.03 | -0.11 – 0.06 | 0.519 |
| Real news sharing intention | 0.63 | 0.57 – 0.69 | <0.001 |
| Condition | 0.05 | -0.06 – 0.17 | 0.366 |
| Observations | 687 | | |
| R^2^ / R^2^ adjusted | 0.399 / 0.397 | | |

H2: The treatment (contrasting to control) will lead to reduced sharing of fake news in the longer run (one-month follow-up).

|  | Fake news sharing intention – Long-term | | |
| --- | --- | --- | --- |
| *Predictors* | *Estimates* | *CI* | *p* |
| (Intercept) | 0.00 | -0.09 – 0.10 | 0.956 |
| Real news sharing intention | 0.58 | 0.52 – 0.65 | <0.001 |
| Condition | -0.01 | -0.14 – 0.13 | 0.938 |
| Observations | 576 | | |
| R^2^ / R^2^ adjusted | 0.339 / 0.337 | | |

H2a: The treatment (contrasting to control) will lead to reduced sharing of fake news immediately after the intervention with control variables.

|  | Fake news sharing intention – Immediate | | |
| --- | --- | --- | --- |
| *Predictors* | *Estimates* | *CI* | *p* |
| (Intercept) | 0.33 | -0.07 – 0.73 | 0.107 |
| Real news sharing intention | 0.54 | 0.47 – 0.60 | <0.001 |
| Condition | 0.05 | -0.08 – 0.19 | 0.441 |
| Age | 0.05 | -0.02 – 0.13 | 0.183 |
| Gender | 0.14 | -0.03 – 0.31 | 0.115 |
| First-generation status | 0.00 | -0.14 – 0.14 | 0.983 |
| Ethnic minority status | -0.49 | -0.85 – -0.12 | 0.009 |
| Cognitive Reflection Task | -0.07 | -0.14 – 0.00 | 0.052 |
| Conspiracy mentality | 0.03 | -0.04 – 0.10 | 0.441 |
| Need for Cognition | -0.01 | -0.08 – 0.07 | 0.867 |
| Digital literacy | -0.00 | -0.08 – 0.07 | 0.916 |
| Observations | 547 | | |
| R^2^ / R^2^ adjusted | 0.340 / 0.328 | | |

H2: The treatment (contrasting to control) will lead to reduced sharing of fake news one month after the intervention with control variables.

|  | Fake news sharing intention – Long-term | | |
| --- | --- | --- | --- |
| *Predictors* | *Estimates* | *CI* | *p* |
| (Intercept) | 0.27 | -0.15 – 0.69 | 0.206 |
| Real news sharing intention | 0.60 | 0.53 – 0.67 | <0.001 |
| Condition | 0.01 | -0.13 – 0.15 | 0.933 |
| Age | 0.05 | -0.03 – 0.12 | 0.252 |
| Gender | -0.11 | -0.29 – 0.07 | 0.245 |
| First-generation status | 0.04 | -0.11 – 0.19 | 0.588 |
| Ethnic minority status | -0.20 | -0.59 – 0.18 | 0.304 |
| Cognitive Reflection Task | -0.03 | -0.11 – 0.05 | 0.432 |
| Conspiracy mentality | -0.01 | -0.08 – 0.06 | 0.772 |
| Need for Cognition | -0.03 | -0.11 – 0.04 | 0.395 |
| Digital literacy | 0.07 | -0.01 – 0.14 | 0.094 |
| Observations | 547 | | |
| R^2^ / R^2^ adjusted | 0.369 / 0.357 | | |
